# One-Year Effectiveness of a 3-Week Balneotherapy Program for the Treatment of Overweight or Obesity

**DOI:** 10.1155/2012/150839

**Published:** 2012-12-24

**Authors:** Thierry Hanh, Patrick Serog, Jérôme Fauconnier, Pierre Batailler, Florence Mercier, Christian F. Roques, Patrick Blin

**Affiliations:** ^1^1 rue Guénégaud, Paris 75006, France; ^2^34 rue d'Eylau, Paris 75116, France; ^3^Pôle Santé Publique, TIMC-IMAG UMR 5525, UJF-Grenoble 1 and CNRS, Themas, 38041 Grenoble, France; ^4^Stat Process, 8 rue de Seine, 27940 Port Mort, France; ^5^Université Paul Sabatier, 118 route de Narbonne, 31000 Toulouse, France; ^6^Service de Pharmacologie, Université Bordeaux Ségalen, INSERM CIC-P 0005, 146 rue Léo Saignat, 33076 Bordeaux Cedex, France

## Abstract

*Objective*. To assess the one-year effectiveness on weight loss of a 3-week balneotherapy program (BT). *Method*. A Zelen double consent randomised controlled trial to compare one-year BMI loss between a 3-week BT program versus usual care (UC) for overweight or obese patients (BMI: 27–35 kg/m^2^), associated or not with a dietary motivational interview (DMI) during the follow-up, using a 2 × 2 factorial design. Main analysis was a per protocol analysis comparing patients attending BT to patients managed by UC, matched on sex, overweight or obese status, DMI randomisation and a propensity score to attend BT or to be managed by UC. *Results*. From the 257 patients who completed the follow-up, 70 patients of each group could be matched. Mean BMI loss was 1.91 kg/m^2^ [95%CI: 1.46; 2.35] for the BT patients and 0.20 kg/m^2^ [−0.24; 0.64] for the UC patients (*P* < 0.001), corresponding to a significant BT benefit of 1.71 kg/m^2^ [1.08; 2.33]. There was no significant effect of DMI and no interaction with BT or UC. No adverse reaction was observed for patients attending BT. *Conclusion*. A 3-week BT program provided a significant one-year benefit over the usual GP dietary advice for overweight and obese patients.

## 1. Introduction

Overweight and obesity correspond to an abnormal or excessive fat accumulation, defined as a Body Mass Index (BMI) ≥ 25.0 kg/m^2^ and ≥30 kg/m^2^, respectively [1]. These chronic situations described as epidemic should be considered as pandemic with a prevalence that has more than doubled since 1980 and which continues to increase dramatically to reach about 1.5 billion overweight or obese people worldwide [1, 2]. Overweight and obesity represent a major risk factor of ischemic heart diseases, ischemic stroke, type 2 diabetes, osteoarthritis, some cancers (endometrial, breast, and colon) [1, 2], and some of the leading diseases in terms of frequency, quality of life impairment, morbidity, mortality, and heath expenses [3].

Public health campaigns and industry-supported changes in our food supply have obviously failed to control the pandemic to date. Many individuals appear to conclude that the benefits of weight management strategies are not worth the cost (i.e., time, money, and continued unrewarding efforts) [4, 5]. This underlines the critical need to implement new, practical, and affordable strategies to complete the armamentarium to fight excess weight.

Balneotherapy (BT), or SPA therapy, is defined as the treatment of disease by bathing, usually at a SPA resort, using hot or cold water rich in minerals, and including also drinking, inhalation, massage through moving water, mud-baths, relaxation, or stimulation [6]. The medical community, mainly in Europe and Asia, has long used BT to improve the symptoms of several chronic diseases, such as osteoarthritis for which recent studies show evidence-based benefit [7–9]. Obesity is another common indication of BT, with preliminary positive results [10–13]. In France, BT has been subsidized by national health insurance for more than 50 years, but scientific evidence to support long-term BT benefit on weight loss has been requested by national health authorities.

Long-term weight control may be facilitated by an appropriate weight loss maintenance strategy such as motivational interviewing, with a weak but significant difference reduction in body mass compared to the control group [14]. Numerous reports have concluded that this modest weight loss contributes to important health benefits [15–17].

The objective of the present study was to assess the one-year effectiveness of a 3-week program of BT for overweight or obese patients and whether a monthly phone dietary motivational interview (DMI) during the follow-up could improve weight loss.

## 2. Methods

The study was a Zelen double consent randomised controlled trial [18, 19] to assess the effectiveness of BT compared to usual care (UC) with or without a monthly phone DMI during follow-up, using a 2 × 2 factorial design. After inclusion by their GP, subjects were equally allocated by a centralized randomisation to one of the following groups: BT alone, BT and DMI, UC alone, and UC and DMI. Zelen double consent was applied to BT randomisation but not to DMI intervention. BT should be done in the 2 months following inclusion. All patients were followed up by the same GP at baseline, 7 months and 14 months after inclusion (i.e., about one year after BT).

### 2.1. Participants

Inclusion criteria were generally healthy adults between the ages of 20 and 70 years consulting their GP for excess weight, with a BMI > 27 kg/m^2^ and ≤35 kg/m^2^. Exclusion criteria were BT contraindication (severe general weakness, inflammatory bowel disease, cirrhosis, severe disability, psychosis and dementia, or immunodeficiency), major eating disorders (bulimia nervosa, compulsive overeating), pregnancy, previous BT for weight problems, poor proficiency, and involvement in another clinical trial. All participants signed an informed consent to participate.

### 2.2. Interventions

BT was a 3-week program in Brides les Bains, Capvern les Bains, Vals les Bains, Vichy, or Vittel BT resorts. The core of the program included 18 daily sessions of (i) individual mineral water bubble bathing at 37°C during 10 minutes; (ii) mineral water manual massages during 10 minutes; (iii) mud body wrap applied at 42°C during 10 minutes; (iv) mineral water pool supervised callisthenic exercises (34°C, 15 minutes); (v) daily dinking of resort mineral water. Mineral waters were mainly sulfate (Brides les bains, Capvern les bains, Vittel) and bicarbonate waters (Vals les bains, Vichy). The specific chemical composition of the five resorts' water is reported in Table 1. Dieticians and personal trainers provided nutrition and physical activity counselling. However, no particular caloric restriction or physical training was mandatory during the patient's stay. Meals were taken freely either at the resort restaurants or anywhere in the town, offering low-fat/low-calorie menu. This program was the usual one for overweight or obese patients, and the staff was not informed which patients were taking part in the clinical trial.

Patients in the UC group received usual weight management advice from the GP consisting of verbal and/or written advice based on the French national guideline, and a brochure “Health comes when eating” [20] was given to the patients at inclusion. Patients were advised to reduce calories, fat, and alcohol, to increase fruit, vegetable, and whole cereal intake, and to incorporate low-intensity, long-duration physical activity into their lifestyle.

DMI was a monthly half-an-hour phone interview with a dietician, consisting in an overview of the current situation, to examine the last 24 hours intake and physical activity, followed by meal composition and food behaviour advice as well as to define 3 or 4 objectives for the next month including at least one about physical activity. DMI started one month after the end of BT for patients attending BT and 3 months after inclusion for UC patients.

### 2.3. Data Collection

Data collected at inclusion were gender, height, birth date, marital status, professional occupation, overweight family history, medical history, weight loss objective, previous drug and nondrug overweight treatment, and drug, examination, dietary, and physical activity prescribed for overweight. Other data collected at inclusion and at the two follow-up visits were the date of visit, weight, body fat mass, waistline measurements, blood pressure and heart rate, physical activity, and tobacco and alcohol consumption. Patient quality of life was assessed using SF12 at inclusion and at the two follow-up visits. Weight was measured by GPs, as well as by a dietician at the end of BT, using the same scale provided for the study.

### 2.4. Outcome

The primary outcome was body mass index (BMI) loss at 14 months of follow-up. Secondary outcomes were weight loss, a weight loss ≥ 5% at 14 months of follow-up and intervention tolerance.

### 2.5. Sample Size

A sample size of at least 139 subjects per group was required to demonstrate a 25% difference between BT and UC groups, with a two-tailed alpha risk of 5%, a power of 90%, and a BMI loss of 1.4 kg/m^2^ (±0.9 kg/m^2^) one year after BT therapy, according to the results of an open pilot study.

### 2.6. Statistics

**Figure 1:**
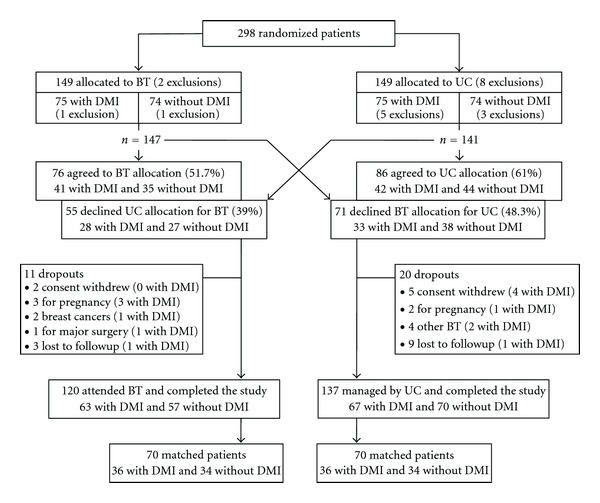
Flow chart of patient recruitment.

According to the Zelen double consent design, a patient could decline the BT or UC randomised allocation to switch to the alternative treatment [18, 19]. This occurred for about half of the patients (Figure 1). With such a complete dilution of the randomisation, it was not relevant to perform intent-to-treat (ITT) analysis. Therefore, in order to reduce a treatment-selection and potential confounding factors as in an observational study, a propensity score to attend BT or to be managed by UC was used. The main analysis compared patients attending BT to optimally matched patients managed by UC. Patients were matched by sex, overweight or obese status, DMI randomisation, and propensity score nearest neighbour with a threshold of 0.1 [21]. A secondary analysis compared all BT and UC patients with DMI stratification and propensity score adjustment.

The baseline comparison between 2 groups used the Student's *t*-test and Chi-square test according to the variables. Propensity scores were estimated using a logistic model with all patient inclusion characteristics. The effect of BT versus UC, DMI versus no DMI, and the interaction between both interventions were assessed using generalized linear model for BMI and weight loss and using logistic model for weight loss ≥ 5% frequency. Adjusted mean and 95% confidence intervals [95%CI] were estimated using Least Squares Mean. A two-tailed *P* value < 0.05 was considered statistically significant. Statistical analysis was performed using SAS software.

The study protocol was ethically approved by the Persons Protection Committee (Paris—Eudract number 2006-A00309-42) and registered to Clinicaltrials.gov number NCT01258114.

## 3. Results

From March 2007 to July 2008, 71 GPs assessed 298 patients, 74 randomised to BT alone, 75 to BT with DMI, 74 to UC alone, and 75 to UC with DMI, of whom 10 were excluded for major exclusion criteria (one with a severe depression, one non-French speaking, and 8 for BMI ≥ 37.5 kg/m^2^). For the 288 remaining patients, 126 (43.8%) declined the randomised allocation to switch to the alternative treatment; 31 dropped out of the study for withdrawal of consent, pregnancy, cancer, major surgery, or other BT during the study or were lost to follow-up (Figure 1). Finally, 257 patients were followed up, 120 patients attending BT (63 randomised DMI), 137 managed by UC (67 randomised DMI, and 70 patients (36 randomised DMI) of each group could be matched (54.5%).

Patients who completed the study had a mean age of 50.9 (SD 11.2) years, 79% were women, 61.1% were obese (≥30 kg/m^2^), 48.6% had a fat mass ≥ 35%, 7.8% were diabetics, 12.8% were smokers, 24.5% were alcohol consumers, 23.3% lived in a large town (≥100 000 inhabitants), 19.8% declared having regular sport activities, and 16.3% had previous treatment for obesity. There were some differences between the 120 patients who attended BT and the 137 managed by UC, whereas the 70 BT and 70 UC matched patients were remarkably similar (Table 2).

**Figure 2:**
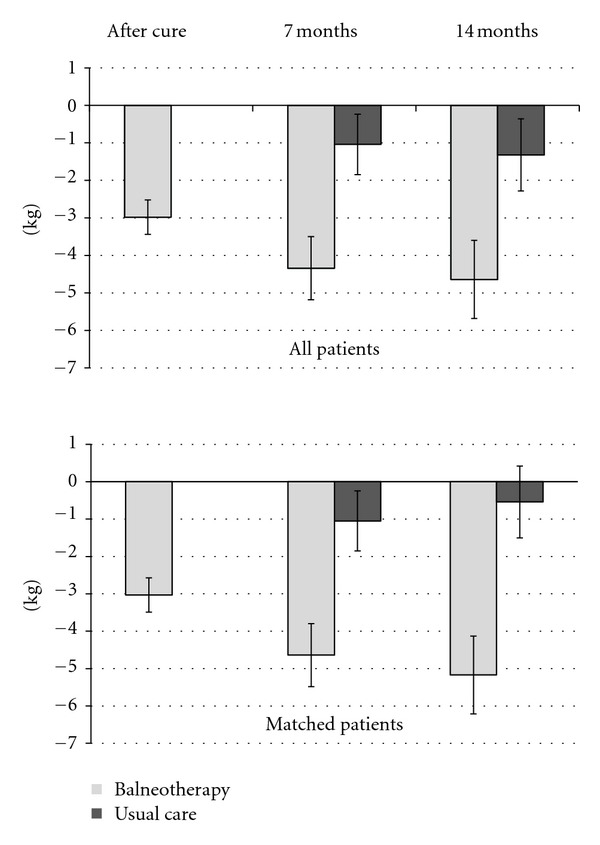
Weight variation (kg) from baseline for matched and all patients.

All patients attending BT completed the 3-week program with a significant mean weight loss of 2.98 kg ([95%CI: 3.44; 2.52], *P* < 0.001) at the end of BT. Then, the benefit continued to improve to 1.47 kg ([0.64; 2.31], *P* = 0.001) from the end of BT to 14 months of follow-up (Figure 2). One-third (32.2%) of the patients reached a weight loss ≥ 5% at the end of BT and half (50.8%) at the end of the study.

Adjusted mean BMI loss at 14 months of follow-up was 1.91 kg/m^2^ [1.46; 2.35] for the 70 BT patients and 0.20 kg/m^2^ [−0.24; 0.64] for the 70 matched UC patients (*P* < 0.001), corresponding to a 1.71 kg/m^2^  [1.08; 2.33] BT benefit. The adjusted mean weight loss was 5.17 kg [3.95; 6.39] and 0.54 kg [−0.68; 1.76], respectively (*P* < 0.001), with a BT benefit of 4.63 kg [2.91; 6.35] (Figure 2). A weight loss ≥ 5% was reached by 40 patients of the BT group (57.1%) and 13 of the UC group (18.6%), with odds ratio of 5.9 ([2.7; 12.8], *P* < 0.001).

For the whole population, BMI and weight adjusted mean loss at 14 months of follow-up were 1.71 kg/m^2^  [1.33; 2.09] and 4.64 kg [3.60; 5.68] for the 120 BT patients compared to 0.50 kg/m^2^  [0.15; 0.85] and 1.32 kg [0.36; 2.28] for the 137 UC patients (*P* < 0.001 for both comparisons). The adjusted mean BT benefit was 1.21 kg/m^2^ [0.65; 1.76] and 3.32 kg [1.80; 4.84]. Results remain significant with the 288 included patients without major exclusion criteria and a Last Observation Carried Forward (LOCF) for the dropout patients (data not shown).

For all analyses, DMI had no significant effect on weight or BMI. There was no significant interaction between the two interventions. No adverse reaction was reported for patients attending BT.

## 4. Discussion

This is the first multicentre clinical intervention trial to examine the one-year effectiveness of BT on weight loss. It shows that a 3-week BT program was associated with significant weight loss at the end of BT and that the benefit continues to improve one year later. The one-year BT benefit, compared to matched UC patients, was 4.6 kg and 1.7 kg/m^2^, with three times more patients reaching a loss of at least 5% of their initial weight. Results were the same when all patients were considered. A monthly phone dietary motivational interviewing (DMI) did not improve this benefit.

The main limit of the study is in relation to the Zelen double consent design. In the review of 58 trials using Zelen design, it was shown that the median of the crossover from one group to the other was 8.9% and interquartile range 2.6% to 15%, which is considered within acceptable limit for an ITT analysis [22]. With about half of patients who crossed over, this study is far from the acceptable limit and overtakes the limit of a Zelen design with a complete dilution of the randomisation (about half of the patients receiving each treatment in both randomized groups). Consequently, this study looks like a prospective nonrandomised clinical intervention trial and it can only be concluded that the weight loss result is more positive for patients who made the choice to go to BT than for those who did not.

Nevertheless, this situation may not be really different from most of the randomised weight management trials where the dropout rate usually reaches 30% to 50% of the patients, generally with different dropout rates between groups [4, 23–26]. The aim of randomisation is to balance the distribution of known and unknown parameters in patients who can receive equally either of the treatments (ambivalence). Double blind maintains the comparability over time. With an open randomised trial and one-third to half of patients dropping out during the follow-up with different dropout rates between groups, can we really assume that the comparability be maintained at the end of the study? Can we also really assume that dropouts keep their last or baseline weight evaluation and do not increase their weight thereafter, as is done with Last Observation Carried Forward (LOCF)? This is the hypothesis underlying ITT analysis. In such a situation, a per-protocol analysis taking into account patient initial differences, as in this study analysis, would probably be more suitable.

In any case, the methodological issue is the validity of the comparison with the control group to define the magnitude of the BT benefit and not the weight loss at the end of BT and one year later. Most primary care physicians believe that weight loss advice and counselling is not a worthwhile activity in clinical practice [2, 27] and they often limit their usual care to verbal or written advice based on national guidelines for healthy lifestyle, without little active follow-up. There is substantial evidence to show that a minimal intervention attitude is ineffective [24, 26, 28–31] and corresponds to the results observed for the weight and BMI variation at one year in our control group managed by usual weight advice from their GP, as well as in the placebo group of most of the randomized trials.

Lifestyle modification programs now represent the cornerstone of treatment for overweight or obesity [30]. The results of our study are compared favourably with those of two reviews on lifestyle modification programs. The first review compared the effect of various diet and activity interventions on weight loss and concluded that lifestyle modification programs induce a weight loss of 4.5% to 6.5% of baseline weight during the year following treatment (16 to 26 weeks of active phase followed by a maintenance phase) [30]. The second review included 80 studies assessing the effect of 8 types of intervention on weight loss (diet alone, diet plus exercise, exercise alone, meal replacement, very low-calorie diet, orlistat, sibutramine, and advice alone) and provided evidence that meal-planning strategies resulted in a mean weight loss of 4.8% to 8% at 12 months [23].

BT benefit is also competitive with commercial weight management programs, for which two recent studies demonstrated a one-year benefit. The first one included 772 overweight and obese patients in Australia, Germany, and UK to compare a commercial program to standard care. For the 61% of patients who completed the 12-month assessment, the weight loss was 6.65 kg in the commercial program group with 3.16 kg benefit from the standard care group [24]. The second one included 740 overweight and obese patients in primary care in the UK to compare 8 weight loss programs of 12 weeks. One year later, 70.5% of the patients were followed up and weight loss ranged from 4.43 kg (2.7; 6.1) to 3.27 kg (1.4; 5.1) for the 3 commercial weight loss program, and 1.26 (−0.6; 3.1) for UC from general practice [26].

Our results also suggest that BT can challenge management of obesity by pharmacological compounds. Indeed, they are surprisingly very similar to those of the latest controlled trial on lorcaserin, a selective serotonin 2C receptor agonist developed for weight loss and recently approved by FDA [32]. This double-blind clinical trial randomly assigned 3,182 obese or overweight adults to lorcaserin, diet, and exercise versus diet and exercise alone; after 1 year, 47.5% of patients in the lorcaserin group lost 5% or more of their body weight compared to 20.3% of patients receiving placebo. Furthermore, BT appears to be a safe intervention in comparison with drugs. Indeed, most drugs developed for weight loss have been removed from the market for severe adverse effects (i.e., fenfluramine, dexfenfluramine, rimonabant, and sibutramine), put under surveillance for severe adverse effects (i.e., orlistat), or discussed for a long time for safety concerns before recent FDA approval (i.e., lorcaserin) [33].

These different data show that the effectiveness of BT on weight loss is similar to the results obtained by drug treatment, commercial weight management programs, and comprehensive lifestyle modification programs. However, a key advantage of a 3-week BT program is the shorter duration of the active phase, which, on average, is 5 to 9 times shorter than other life style modification programs. Shorter programs are more readily acceptable and better tolerated [4], with a reduction of the economic burden that appears to be correlated with the duration of the program [34]. Attrition rates attributable to lifestyle modification programs are above 35% in one-third of the clinical trials [31] and appear to be strongly correlated with the treatment duration [4]. Conversely, all patients who started BT completed this intervention.

This study does not allow the individualization of the effectiveness of different compounds of the 3-week program: water care, water drinking, lifestyle modification from nutrition or physical activity counselling, and 21 days of peace and quiet outside the usual home. However, a study involving 30 patients showed that the two weeks BT have an impact on the plasma level of the two adipocytokines leptin and adiponectin: a slight but not significant increase of leptin which regulates the appetite and energy at the hypothalamic level and a significant decrease of adiponectin, whose main action is to improve insulin sensitivity [35, 36]. The relationship between weight loss and adipocytokine variations after BT needs further investigation.

## 5. Conclusion

BT is an efficient lifestyle modification program, which provides significant weight loss and long-term maintenance with only 3 weeks of intervention. BT appears to be an effective and safe program that can be used by primary care physicians as a first-line treatment option for overweight and obese patients.

## Figures and Tables

**Table 1:** Chemical composition of the water of five resorts.

Balneotherapy resorts	Main ions (mg/L)											
	Ca	Mg	Na	K	SO_4_	Cl	HCO_3_	Ca	Mg	SiO_2_	Other ions	CO_2_ (gas)
Brides les bains	634	104	1365	89.8	2720	1413	427	41.8	634	104	F, Li, Sr	No
Capvern les bains	335	64.7	5.0	1.4	984	5.0	107	8.7	335	64.7	Sr	No
Vals les bains	60.0	46.5	2275	—	20.3	126	6196	—	60.0	46.5	—	yes
Vichy	113	12.4	1909	89.7	178	369	4709	63	113	12.4	F, Li, Fe, As	yes
Vittel	575	118	12.5	4.7	1584	7.0	377	7.6	575	118	F, Li, Sr	No

**Table 2:** Patient characteristics at baseline.

	All patients			Matched patients		
	Balneotherapy	Usual care	*P*	Balneotherapy	Usual care	*P*
	*n* = 120	*n* = 137		*n* = 70	*n* = 70	
Gender, *n* (%)			0.54			1.0
Male	23 (19.2%)	31 (22.6%)		11 (15.7%)	11 (15.7%)	
Female	97 (80.8%)	106 (77.4%)		59 (84.3%)	59 (84.3%)	
Age—years, mean ± SD	51.3 ± 11.5	50.5 ± 10.9	0.54	52.1 ± 11.3	51.9 ± 10.8	0.90
Married or living with a partner, *n* (%)	78 (65.0%)	99 (72.3%)	0.23	42 (60.0%)	48 (68.6%)	0.38
Number of children, *n* (%)			0.83			0.84
None	22 (18.3%)	28 (20.4%)		16 (22.8%)	13 (18.6%)	
One	23 (19.2%)	22 (16.1%)		13 (18.6%)	13 (18.6%)	
≥2	75 (62.5%)	87 (63.5%)		41 (58.6%)	44 (62.8%)	
Living in town ≥ 100.000 population, *n* (%)	23 (19.2%)	37 (27.0%)	0.14	13 (18.6%)	17 (24.3%)	0.54
BMI—kg/m^2^, mean ± SD	31.3 ± 2.7	31.2 ± 2.5	0.76	31.4 ± 2.8	31.3 ± 2.6	0.75
Weight status, *n* (%)			0.70			1.0
Overweight	75 (62.5%)	82 (59.9%)		42 (60.0%)	42 (60.0%)	
Obese	45 (37.5%)	55 (40.1%)		28 (40.0%)	28 (40.0%)	
Fat mass > 35%, *n* (%)	64 (53.3%)	61 (44.5%)	0.17	38 (54.3%)	40 (57.1%)	0.87
Waistline measurement >100 cm, *n* (%)	73 (60.8%)	86 (62.8%)	0.80	46 (65.7%)	45 (64.3%)	1.0
Paternal history of obesity, *n* (%)	46 (38.3%)	44 (32.1%)	0.36	24 (34.3%)	28 (40.0%)	0.60
Maternal history of obesity, *n* (%)	59 (49.2%)	58 (42.3%)	0.32	34 (48.6%)	32 (45.7%)	0.87
Sibship history obesity, *n* (%)	40 (33.3%)	45 (32.9%)	1.0	25 (35.7%)	25 (35.7%)	1.0
Diabetic, *n* (%)	14 (10.2%)	6 (5.0%)	0.16	8 (11.4%)	4 (5.7%)	0.37
Current smoker, *n* (%)	12 (11.7%)	21 (15.3%)	0.26	7 (10.0%)	9 (12.9%)	0.79
Regular alcohol consumption, *n* (%)	29 (24.2%)	34 (24.8%)	1.0	17 (24.3%)	11 (15.7%)	0.29
Regular physical activities, *n* (%)	23 (19.2%)	28 (20.4%)	0.88	14 (20.0%)	11 (15.7%)	0.66
SBP ≥ 130 mmHg, *n* (%)	81 (67.5%)	70 (51.1%)	<0.01	44 (62.9%)	44 (62.9%)	1.0
DBP ≥ 80 mmHg, *n* (%)	75 (62.5%)	70 (51.1%)	0.08	44 (62.9%)	41 (58.6%)	0.73
Pulse ≥ 70 beats per minute	65 (54.2%)	53 (38.7%)	0.02	36 (51.4%)	32 (45.7%)	0.61
SF12 mental score, mean ± SD	59.3 ± 1.1	59.4 ± 1.1	0.44	59.4 ± 1.1	59.2 ± 1.1	0.26
SF12 physical score, mean ± SD	44.6 ± 9.8	46.5 ± 9.2	0.16	44.0 ± 10.4	46.5 ± 9.8	0.18
